# Transcranial Magnetic Stimulation in Disorders of Consciousness: An Update and Perspectives

**DOI:** 10.14336/AD.2022.1114

**Published:** 2023-08-01

**Authors:** Weilong Huang, Qiang Chen, Jun Liu, Lin Liu, Jianhong Tang, Mingang Zou, Tianxiang Zeng, Huichen Li, Qing Jiang, QiuHua Jiang

**Affiliations:** ^1^Department of Neurosurgery, Ganzhou People's Hospital, Jiangxi, China; ^2^Key Laboratory of Prevention and Treatment of Cardiovascular and Cerebrovascular Diseases of Ministry of Education, Gannan Medical University, Jiangxi, China; ^3^Laboratory Animal Engineering Research Center of Ganzhou, Gannan Medical University, Jiangxi, China

**Keywords:** disorders of consciousness, repetitive transcranial magnetic stimulation, treatment, patients, clinical

## Abstract

Disorders of consciousness (DOC) is a state in which consciousness is affected by brain injuries, leading to dysfunction in vigilance, awareness, and behavior. DOC encompasses coma, vegetative state, and minimally conscious state based on neurobehavioral function. Currently, DOC is one of the most common neurological disorders with a rapidly increasing incidence worldwide. Therefore, DOC not only impacts the lives of individuals and their families but is also becoming a serious public health threat. Repetitive transcranial magnetic stimulation (rTMS) can stimulate electrical activity using a pulsed magnetic field in the brain, with great value in the treatment of chronic pain, neurological diseases, and mental illnesses. However, the clinical application of rTMS in patients with DOC is debatable. Herein, we report the recent main findings of the clinical therapeutics of rTMS for DOC, including its efficacy and possible mechanisms. In addition, we discuss the potential key parameters (timing, location, frequency, strength, and secession of rTMS applications) that affect the therapeutic efficiency of rTMS in patients with DOC. This review may help develop clinical guidelines for the therapeutic application of rTMS in DOC.

## 1. Introduction

Disorders of consciousness (DOC) is a state in which consciousness is affected by brain injuries, leading to dysfunction in vigilance, awareness, and behavior [1]. The main DOCs are coma, unresponsive wakefulness syndrome (UWS), formerly termed as a vegetative state (VS), and minimally conscious state (MCS). Their distinction is often described in two aspects: wakefulness and awareness. Wakefulness, a state of arousal, refers to the level of consciousness (deep sleep, drowsiness, and normal wake) that can be measured by the existence of eye-opening and brainstem responses. Awareness refers to the content of consciousness, which includes the ability of an individual to respond to both external and internal stimuli in an integrated manner [2, 3]. A patient in coma has no signs of being awake and aware and does not respond to their environment, voices, or pain with closed eyes. Comatose patients may remain in that state for 2 to 4 weeks, during which time they may begin to recover consciousness or enter a VS/UWS or MCS [4]. VS/UWS is characterized by wakefulness but no signs of awareness, such as no meaningful responses or signs of experiencing emotions. However, patients with VS/UWS have basic reflexes, can open their eyes, and can regulate their heartbeat and breathing without assistance [5]. Therefore, the VS/UWS is a state in which basic vegetative nervous functions, such as thermoregulation, respiration, and sleep-wake cycles are preserved, but with a complete deficiency of sensation or thought. However, recent evidence indicates that several characteristics of cortical function, including somatosensory, nociceptive, auditory, and semantic processing, can persist even without consciousness [6]. When this situation lasts over four weeks, it is referred to as a continuing VS/UWS, after which the chances of recovery decrease with time. When VS/UWS lasts longer than 12 months after traumatic brain injury (TBI) and beyond 3 months (6 months is also suggested) after non-TBI, it is referred to as a persistent vegetative state (PVS), which was proposed by the Multi-Society Task Force to classify the irreversibility of the condition [7]. PVS is thus considered to indicate the irreversibility of the condition, but it is not impossible [8]. Some patients with VS/UWS recover a certain degree of awareness, thus progressing to MCS [9], which is described as being awake and able to show, if intermittently, reproducible signs of awareness in the form of purposeful behavior. Patients in MCS can be subclassified into MCS *plus* (MCS+) and MCS *minus* (MCS-), depending on the complexity of the behavioral responses: intelligible verbalization or intentional communication. MCS *minus* is defined as non-communicative responses to meaningful stimuli. The most common symptoms of consciousness in patients with MCS *minus* are visual fixation and pursuit, automatic motor reactions, and localization to noxious stimulation [10], whereas MCS *plus* is characterized by intelligible verbalization or intentional communication [11]. Locked-in syndrome is not a DOC but a rare neurological disorder; however, it can appear to be caused by paralysis of all voluntary muscles except for those that control eye movements [12].

As described above, patients with DOCs can be differentially diagnosed based on their level of wakefulness and awareness. Although severity may differ from patient to patient, lack of overt behavior is the common denominator of patients with DOC. The diagnostic criteria, such as the Glasgow Coma Scale and JFK Coma Recovery Scale-Revised (CRS-R), for assessing the degree of consciousness in patients with DOC created by the Aspen Workgroup and their guidelines have proven to be valuable clinical tools; however, some limitations remain [9]. Indeed, the CRS-R meets all Aspen Workgroup criteria that have been used as a standardized neurobehavioral assessment measure for patients with DOC, but false negative diagnostic errors occur [13]. However, diagnosis of DOC based on a purely behavioral assessment is also challenging because it is difficult to study neural processes that are not linked to or may be disconnected from behavior [14]. Therefore, accurately diagnosing patients with DOC remains challenging, and misdiagnosis can occur when differentiating patients in a VS/UWS from those in an MCS, as no method exists that can directly determine one’s consciousness [15]. The distinction between “conscious” and “unconscious” depends on the pragmatic principle by which the presence of voluntary behavior is taken to indicate the occurrence of consciousness [16]. Forty-one percent of MCS cases have been allegedly misdiagnosed as UWS. This emphasizes the need for improved diagnostic technologies [17]. To improve diagnostic accuracy for patients with DOC, neuroimaging and electrophysiological tools, including positron emission tomography (PET), functional magnetic resonance imaging (fMRI), electroencephalography (EEG), and transcranial magnetic stimulation (TMS), have been proposed to help assess brain activity and the level of brain damage [18, 19]. Importantly, the diagnosis cannot depend on a single assessment. To improve the accuracy of behavioral measurements in differentiating VS/UWS and MCS patients, advanced neuroimaging and electrophysiological technologies may be incorporated into standard clinical practice, although the patients do not necessarily show awareness [20, 21]. In summary, the diagnosis of DOC is methodologically complex and requires careful interpretation.

DOC is generally caused by severe injuries to the brain regions that are involved in regulating consciousness. The main causes are TBI and non-TBI conditions, such as stroke and heart attacks, and progressive brain damage, such as Alzheimer's disease. Establishing the cause of brain injury is critical for a reliable diagnosis of DOC. According to the Centers for Disease Control and Prevention (CDC) database (www.cdc.gov/traumaticbraininjury), TBI is a major cause of death and disability in the United States. Annually, nearly 1.5 million people sustain TBI in the United States, and 80,000-90,000 people live with TBI-related disabilities. Globally, over 27 million new cases of medically treated TBI occurred in 2016, with an age-standardized incidence of 369 per 100,000 people worldwide [22, 23]. Nearly 0.3% of severe TBIs can cause DOC [24]. Therefore, the number of patients with DOC has been rapidly increasing worldwide. DOC not only impacts the lives of individuals and their families but is also becoming a serious public health threat.

Unfortunately, although our understanding of DOC has progressed remarkably in recent years, uncertainty remains with respect to standard treatment, although care recommendations for patients with DOC have been proposed [19, 25]. For example, no consensus treatment guidelines are available for patients with DOC, and few interventions have proven any level of progress. Currently, therapeutic options include pharmacological and non-pharmacological treatments. Pharmacological treatments include dopamine agonists and N-methyl-d-aspartate antagonist dopaminergic agents (levodopa and amantadine), dopamine receptor agonists (bromocriptine), and gamma-aminobutyric acid (GABA) agonists (zolpidem) [26], whereas non-pharmacological treatments include median nerve stimulation or surgical management using deep brain stimulation, extradural cortical stimulation, and spinal cord stimulation [27, 28]. These interventions aimed to improve the patient’s level of consciousness [29]. However, several randomized controlled trials have suggested that only two studies on amantadine and transcranial direct current stimulation provided class II evidence [29]. Repetitive TMS (rTMS) is a non-invasive and efficient brain function regulation technology that can stimulate electrical activity by a pulsed magnetic field in the brain and has great potential value in the treatment of chronic pain, depression, neurological diseases (Alzheimer’s disease), and mental illnesses [30]. However, the clinical applications of rTMS in patients with DOC remain debatable [31, 32].

In this review, we report the recent main findings of the clinical therapeutics of rTMS for DOC, including its efficacy and possible mechanism. In addition, we discuss the key parameters (timing, location, frequency, strength, and secession of rTMS applications) that affect the therapeutic efficiency of rTMS in patients with DOC.

## 2. Clinical applications of TMS

### TMS and rTMS

TMS is a noninvasive and painless brain stimulation device that can modulate brain excitability and networks [33] and relies on electromagnetic induction by a short-lasting current through an insulated coil. The coil produces brief magnetic pulses that pass easily and painlessly through the skull and into the brain when placed tangentially on the head. Therefore, TMS interconverts electrical and magnetic energy to induce electromagnetic events that can depolarize cortical neurons [34]. TMS can be used as one stimulus at a time (single-pulse TMS), in pairs of stimuli separated by a variable interval (paired-pulse TMS), or in trains (rTMS) [35]. rTMS consists of repeated single-pulse stimulation delivery to specific brain areas [36], which can produce long-lasting changes in brain activity, thereby altering cortical excitability [37]. Therefore, TMS or rTMS is considered a non-invasive technique, as the electromagnetic coil is placed on the scalp and thus does not require craniotomy or seizure induction to make its electrical stimulation electrodeless [38].

### Clinical applications of rTMS

TMS was developed by Barker et al. in 1985 [39] and showed a motor-evoked potential response when placed tangentially on the head [39]. TMS was initially used to stimulate the neurons in the brain to improve the symptoms of depression. rTMS was first applied to patients with depression only a few years after the technology for repetitive stimulation was developed. To date, rTMS has been used for many psychiatric diseases, such as addiction, dementia, major depressive disorder (MDD), obsessive-compulsive disorder (OCD), and schizophrenia [40, 41]. rTMS has been proposed to alleviate symptoms in patients with stroke, neuropathic pain, Parkinson’s disease, and other neurological disorders [30]. Studies have documented the therapeutic efficacy of rTMS in depression, pain, stroke-mediated motor deficits, and schizophrenia [42]. The US Food and Drug Administration (FDA) authorized the first rTMS device for the treatment of MDD patients with poor response to at least one pharmacological agent in 2008 and for the treatment of OCD using deep TMS with an H7-coil in 2018 [40]. In 2014, a group of European experts established guidelines on the therapeutic application of rTMS according to the evidence published in 2014 [42]. In 2020, the guidelines were reappraised based on evidence published between 2014 and 2018 [30].

In the 2014 guidelines, the recommendation included pain, movement disorders, stroke, amyotrophic lateral sclerosis, epilepsy, multiple sclerosis, tinnitus, consciousness disorders, depression, OCD, anxiety disorders, schizophrenia, craving/addiction, and conversion [42]. However, the DOC was not included in this study. In the 2020 guidelines, although a clinical benefit on the level of consciousness is described after rTMS treatment, a small sample number is found in all published papers, indicating the shortage of high-quality studies. Therefore, the committee did not propose any level of evidence or make recommendations for the use of rTMS in patients with chronic DOC [30]. It is necessary to determine how to optimize rTMS protocols for the treatment of patients with DOC in routine clinical practice.

## 3. The therapeutic efficacy of rTMS in patients with DOC

During the last decade, the clinical use of rTMS in patients with DOC has been attempted, while the therapeutic efficacy of this therapy remains largely unknown [1, 43-47]. In a recent randomized, double-blinded, sham-controlled trial, 40 DOC patients received either active-rTMS or sham-rTMS 49.0 ± 24.6 days after the onset of DOC [31]. The active-TMS protocol consisted of a frequency of 20 Hz (trains of 20 pulses over 1βs, at intervals of 20βs; 20 sessions; 5 times a week) for consecutive week intervention on the left dorsolateral prefrontal cortex (DLPFC) with a 100% resting motor threshold. Consciousness was evaluated using the CRS-R, which is the strongest recommendation of the American Congress of Rehabilitation Medicine for behavioral assessment scales detecting consciousness in patients with subacute-to-chronic DOC [48]. One session of the rTMS procedure includes 2,000 pulses (20βHz, trains of 20 pulses over 1βs, at intervals of 20βs) at an intensity of 100%. As expected, no significant difference in the CRS-R scores was found between the two groups before rTMS application. Compared to sham-rTMS (6.25 ± 1.29), patients with DOC in the active rTMS treatment with a frequency of 20βHz had markedly improved consciousness (8.45 ± 3.55). However, an in-depth analysis revealed that only 50% of patients with DOC exhibited improvement in consciousness and responded to rTMS. Furthermore, rTMS did not considerably increase the awakening ratio [31]. The data collected from the analysis suggests the therapeutic efficacy of rTMS in improving consciousness in some patients with DOC. Furthermore, in a recent randomized pseudo-controlled study, high-frequency rTMS or false rTMS stimulation of DLPFC was used in 48 patients with PVS for 60 days [49]. After 30 days of rTMS treatment, the CRS-R scores of the two patients with PVS improved, but the difference in clinical behavior was not significant. Similarly, the CRS-R scores in the control group were not significantly different after 30 days of treatment. After 60 days of treatment, the CRS-R scores of 12 patients with PVS in the treatment group improved, suggesting that rTMS improves the recovery of consciousness impairment [49]. In contrast, Cincotta et al. [45] (N = 11 VS/UWS patients) and Liu et al. [50] (N = 7 DOC patients) tested 20 Hz rTMS of the primary motor cortex (M1) for five consecutive days but found no evidence of a therapeutic effect (improved CRS-R and Clinical Global Impression-Improvement [CGI-I] scores). Using a similar protocol, He et al. [51] found that 5 patients with DOC showed localized brain reactivity, but only one patient showed long-lasting behavioral and neurophysiological changes.

In another comparable study [52], 16 patients with chronic DOC (5 MCS and 11 UWS) were treated with active 10 Hz rTMS (one session per day for 20 consecutive days) on the right DLPFC. A single session of stimulation comprised 1,000 pulses (10 Hz; repeated 10 times with an inter-train interval of 60 s; and 11 min and 40 s for the total session). The outcome was determined using the CRS-R and CGI-I scores. The CRS-R scores of five MCS patients and four of 11 VS/UWS patients significantly improved compared to the baseline in all participants. Interestingly, the improvement was more remarkable in patients with MCS than in those with VS/UWS. The CGI-I scores of two patients increased significantly, two improved, six minimally improved, six experienced no change, and none worsened. Application of 10 Hz multisession rTMS on the right DLPFC can improve the consciousness state of patients with DOC, increase the CRS-R score, and decrease motor evoked potential (MEP) latency and central motor conduction time (CMCT), especially in MCS [52].

Similarly, Ge et al. stimulated the right DLPFC of 15 VS/UWS patients with 10 Hz rTMS [53] and found that the CRS-R scores were significantly increased compared with pretreatment scores after 20 days of treatment. Of these, seven VS/UWS patients had improved CRS-R scores by over 4 points in the rTMS group, but only two control subjects showed a CRS-R score improvement by 3 points. Most patients showed no significant increase in CRS-R scores. The average CRS-R score was 3 points in the rTMS group but only 1 point in the control group. Thirteen patients with UWS (86.7%) and only 5 patients (29.4%) in the control group received rTMS turned MCS. This data indicates that rTMS application can improve patient awareness and accelerate VS/UWS patient recovery [53]. However, in another pilot study, 10 VS/UWS patients who received a single session of 10 Hz rTMS on the right DLPFC did not show any clinical improvement or intra- or intercortical connectivity changes at the group level [46].

Generally, patients in an MCS are more likely to respond to rTMS than those in a PVS (71.4 vs. 22.9% overall). For example, previous studies have shown that 83.3% of MCS and 45.5% of PVS patients after stroke are more likely to respond to rTMS [54]. Studies have also shown that eight and nine MCS patients, but no PVS patients, demonstrated improvement in the Speech/Vocuity or arousal CRS-R subscore domains. Over 50% of patients with MCS had improved visual subscores (19/30; 63.3%), whereas PVS patients most likely improved in the motor subscore domain (8/11; 72.7%) [54].

Recently, O'Neal et al. performed a meta-analysis of individual patient data (90 patients with DOC) to examine the effects of studies utilizing TMS on the outcomes of patients with DOC [54]. They found that TMS, rTMS in particular, could improve the CRS-R scores of patients with DOC after one session of TMS and at the last post-TMS CRS-R assessment [54]. Patients with DOC who received TMS had significantly higher post-TMS CRS-R scores than pre-TMS CRS-R scores for all assessed post-TMS time points [54].

## 4. Mechanisms of rTMS for improvement of DOC

Although rTMS has been considered a promising therapeutic tool for patients with DOC, the mechanism underlying the improvement of consciousness in patients with DOC remains largely unknown. The potential mechanisms may include the following: (1) modifies neural circuits and brain networks, (2) promotes synaptic plasticity, (3) restores neurotransmitters, (4) increases neural stem cell proliferation, and (5) increases neurotrophins. The effects may be beneficial for improving adverse outcomes in patients with DOC.

TMS can generate a magnetic field, thereby inducing depolarization of neural cell membrane potentials in the cortical region under the coil and impacting the related nerve loop activity, which can be detected by EEG, a well-known and widely used technique for the visualization of brain activity. Therefore, rTMS-mediated cortical stimulation may activate, inhibit, or interfere with the activity of neural networks depending on the stimulus frequency, location, and intensity [55]. For example, the excitability of the motor cortex could be decreased by low-frequency-rTMS administration, but increased by high-frequency-rTMS administration [56]. Estradiol has a strong influence on cortical excitability. Serum estradiol levels could be affected by high-frequency-rTMS and are positively related to clinical responses in male patients with DOC [57]. Importantly, the electric field induced by rTMS affects not only the activity of cortical circuits but also affects those fibers that project antidromically or orthodromically to distant brain structures, as well as the excitability of white matter structures. Therefore, although it is known that rTMS activates neurons in the cortex rather than in deeper parts of the brain, in addition to the target regions, rTMS also activates distant interconnected sites in the brain, which is important for conveying interactions between different brain areas involved in brain networks [31].

Indeed, rTMS can increase functional connectivity via synaptic plasticity, such as long-term potentiation/ depression (LTP/LTD) of excitatory synaptic transmission [58], through altered gene and protein expression involved in N-methyl-D-aspartate (NMDA) receptor function [55, 59]. LTP and LTD may be crucial mechanisms supporting long-term changes in synaptic strength after TMS and are crucial processes for enhancing functional networks. Studies have reported that high-frequency-rTMS could improve cortical excitability and induce a profound effect on neuroplasticity markers, such as the glutamate receptor 1 (GluR1) subunit of the α-amino-3-hydroxy-5-methy-4-isoxazole propionate (AMPA) receptor [60]. However, low-frequency-rTMS (≤ 1 Hz) reduces synaptic efficiency [60, 61]. Glutamate transmission plays a fundamental role in LTP and LTD, and the role of AMPA receptors in the mechanism of rTMS has been suggested [62].

In addition to neuroplasticity, rTMS can restore neurotransmitters. For example, rTMS is accompanied by changes in GABA, a major inhibitory neurotransmitter in the brain, in local hippocampal inhibitory circuits [63]. Serotonin (5-HT) is a vital excitatory transmitter. High-frequency-rTMS sessions on the left DLPFC could upregulate left hippocampal baseline 5-HT(2A) receptor binding indices (BI) but reduce right hippocampal 5-HT(2A) receptor uptake values in patients with depression [64]. Several studies have shown that prefrontal rTMS can accelerate dopamine release in the mesostriatal, mesolimbic, and striatal regions, as well as glutamate and gamma-aminobutyric acid levels [65]. Therefore, dopaminergic treatment may lead to enhanced improvement in patients with DOC.

rTMS has neurotrophic effects on dendritic growth, sprouting, and neurotrophic factors by increasing neurotrophins such as brain-derived neurotrophic factor (BDNF). These factors are essential for the survival, differentiation, and regeneration of neurons [66], as well as synaptic transmission and plasticity. Several studies have reported that rTMS with low-intensity stimulation (110% average rMT, 1 Hz) triggered the activation of BDNF/tropomyosin-related kinase B (TrkB) and upregulated the levels of synaptic protein markers, such as growth-associated protein 43 (GAP43), synaptophysin (SYN), and post-synaptic density protein 95 (PSD95), as well as decreased synapse density and post-synaptic density (PSD) thickness [66]. In contrast, high-intensity magnetic stimulation (150% rTM, 1 Hz) appeared to be detrimental, inducing thinning of PSDs, disordered synaptic structures, and a large amount of lipofuscin accumulation, as well as reducing the number of synapses and downregulating BDNF-TrkB and synaptic proteins.

Increased neurogenesis in the hippocampus is also suggested to be a therapeutic mechanism of rTMS [67, 68]. Studies have reported that rTMS (25 Hz rTMS for 2 weeks) increased neurogenesis in the hippocampal dentate gyrus in rats [65] and stimulated neural stem cell proliferation in vitro [69]. Although the underlying mechanism remains unknown, rTMS-induced upregulation of growth factors such as BDNF may be crucial for increased hippocampal cell proliferation and neuronal differentiation after rTMS application [70]. A recent study reported that 20 Hz rTMS significantly promoted neurogenesis, along with elevated protein levels of BDNF and phosphorylated-TrkB [71].

Taken together, these effects may be beneficial in ameliorating adverse outcomes in patients with DOC. However, the precise mechanisms by which rTMS improves consciousness remain poorly understood, and mechanism-based treatments for DOC remain elusive. By understanding the mechanism of action of rTMS on the brain, we can assess the potential for therapeutic practical application of TMS in clinical settings.

## 5. Key parameters influencing the therapeutic efficacy of rTMS

The stimulation parameters of rTMS, including stimulation frequency, stimulation intensity, stimulation location (target regions), coil direction, stimulation pattern, stimulation interval and duration, and stimulation sessions are related to the biological effects of rTMS and thus significantly impact the therapeutic efficiency of rTMS in patients with DOC [72]. Different combinations of stimulation parameters can have different biological effects [73]. However, the optimal stimulation parameters that are critical for rTMS application in clinical settings remain unclear, as different parameters of rTMS may activate, suppress, or interfere with the activities of neural cells in different cortical areas, thus leading to different therapeutic effects [74].

### Timing

Time may be an important parameter for evaluating the outcomes after rTMS application. First, some MCS and PVS patients may recover on their own with time and thereby functional outcome is significantly more favorable for MCS patients relative to UWS patients [13, 75, 76]. Modifications in the CRS-R scores may depend on rTMS rather than time. In addition, MCS *plus* patients have greater metabolic activity and resting-state functional connectivity in the language network [15, 77], suggesting that the consciousness state is the key factor that affects outcome evaluation. Second, patients in the acute, subacute, and chronic stages of DOC may have different responses to rTMS. Although the data are limited, DOC patients showed significantly greater CRS-R scores if rTMS was initiated < 3 months after injury compared to those in whom rTMS was initiated 3 months after injury [54]. Consistent with this finding, Angelakis et al. reported that multiple repeated sessions of TMS may improve the clinical status of MSC patients who have been in this condition for < 12 months [78]. Therefore, patients in the acute stage of DOC have better outcomes than those in the subacute and chronic stages of DOC after initiating TMS, and the time between inciting injury and TMS initiation is an important factor. The therapeutic efficacy of rTMS treatment should be carefully interpreted.

### Location

The magnetic field easily penetrates the skull and induces electrical currents in the brain regions under the coil with a penetration depth of approximately 3 cm [79]. However, it changes rapidly, from zero to a very high value, and then returns to zero again in 1 ms. Therefore, the position and location of rTMS are critical for functional outcomes [33], as (1) rTMS mainly acts on cerebral cortex cells (neurons and astrocytes), while the brain areas located deep below the skull remain unrealistic to target at present [72], and (2) the brain anatomical structure linked to consciousness and performing conscious activity includes the wide cerebral cortex and ascending reticular activating system. Therefore, it is critical to select the cortical position for rTMS for boosting consciousness improvement. The US FDA approved the use of prefrontal TMS therapy repeated daily for 4-6 weeks for the treatment of patients with MDD resistant to medications [80]. However, the site of stimulation for DOC varied in previous studies, as there is no evidence to clearly demonstrate the superiority of the stimulation sites [31]. The primary motor cortex (M1) [1, 44, 45] and the DLPFC [43, 46] are the most commonly chosen stimulation sites, as the DLPFC plays an important role in motor control and behavioral function, as well as in the decision-making network [81]. The DLPFC is thought to enhance learning, memory, and attention, whereas attention, memory, and consciousness have the closest correlation with awareness [82, 83]. Studies have consistently documented that stimulation of the M1 could improve motor impairment after stroke or cognitive function [78, 84, 85], or after cognitive function in verbal fluency and working memory in healthy volunteers [86-89]. Similarly, magnetic stimulation of the DLPFC improves learning and memory [82]. However, 50.7% of patients with TMS targeting non-M1 cortical regions showed a response to TMS compared to those receiving TMS targeting M1 regions (30.4%), even if the stimulation location was not significant based on a linear regression model [54].

Interestingly, the right and left DLPFC as the site of stimulation could result in different clinical outcomes. For example, a study reported that negative nonaffective switch costs in healthy women significantly improved immediately after a single session of left DLPFC rTMS, but not after a session of right DLPFC rTMS [90]. The right DLPFC has been connected to the upkeep of sustained arousal and attention because it has strong connections with reticular formation [91], which is similarly relevant for patients with DOC. One study reported that a single session of 10 Hz rTMS over the right DLPFC did not induce clinical improvement or intra- or intercortical connectivity modifications [46]. The left DLPFC region receives visual and somatosensory inputs from the parietal heteromodal association cortices involving vision, motion, spatial orientation, and tactile sensations and projects to subcortical, monoaminergic, and cholinergic sources [92]. In addition, the left DLPFC is involved in stimulus-specific information processing for encoding episodic memories, suggesting a role in memory formation [93]. There is evidence that high-frequency rTMS over the left DLPFC improves the linguistic and cognitive skills of patients with Alzheimer’s disease [94] and reduces the symptoms of major depression in Parkinson’s disease [95]. This approach has been selected for a range of transcranial direct current stimulation applications in patients with DOC [78, 92]. Stimulating the left DLPFC with slow rTMS leads to enhanced word memory performance [96]. Therefore, the choice of site as the stimulation target may be based on the function of the brain region of stimulation. Although various stimulation sites have been used, relatively few studies have used right-sided stimulation. Therefore, it remains difficult to determine the effects of different stimulation sides on changes in the CRS-R index. Interestingly, in the postacute stage after stroke, most studies use the ‘‘unaffected” contralesional motor cortex for the delivery of stimulation [30].

Taken together, the stimulation location is important for clinical applications. Before rTMS is applied, the location of the brain damage should be identified for better clinical outcomes. TMS modalities, including bilateral stimulation, deep TMS, and theta burst stimulation, may be considered in the treatment of patients with DOC.

### Frequency

Frequencies ranging from 1 Hz to 25 Hz have been used in clinical settings. The excitability of neuronal stimulation levels can be modified by either high (10-20 Hz) or low (1 Hz) frequency. Therefore, TMS modalities include high-frequency (≥ 5βHz) and low-frequency (≤ 1βHz) stimulations. Despite the intra- and inter-individual variability of responses to rTMS [97], low-frequency rTMS could inhibit cortical excitability, while high-frequency rTMS may induce excitatory impacts [98]. Low- and high-frequency rTMS protocols show a strong dichotomy such that the low-frequency decreases cortical excitability to form long-term depression, whereas the high-frequency produces accelerating oscillatory activity to form long-term potentiation [31]. High-frequency stimulation of the left DLPFC relieves depressive symptoms, whereas low-frequency cortical stimulation of the right DLPFC alleviates the symptoms of both depression and anxiety. High-frequency rTMS (10βHz or 20βHz) is often performed to treat patients with DOC and other neurological disorders [51, 52]. For example, He et al. conducted a randomized, sham-controlled, crossover study to treat six patients with DOC for five consecutive days using real or sham 20 Hz rTMS, which was applied to the left M1 [51]. They found that only one patient with a 2-month history of TBI showed behavioral changes and EEG modifications after real rTMS stimulation. The remaining subjects did not show any significant behavioral effects or overall EEG modifications [51]. In contrast, another study reported that 20 Hz rTMS to the M1 for five consecutive days (1,000 pulses/session) in 11 patients with VS did not significantly improve outcomes based on the CRS-R and CGI-I scales and EEG modifications [45]. As the number of studies is limited, it remains unknown whether the typical 10 Hz high-frequency stimulation protocol is better than 20 Hz frequency stimulation for the treatment of patients with DOC.

The H coil can stimulate deeper and broader regions than the standard rTMS using a figure-8 coil, and thus can be used for deep TMS [99], which has been approved by the FDA for clinical application. Deep TMS has been proven efficacious for the treatment of various disorders, such as depression, chronic tinnitus, digital pain, and posttraumatic stress disorder [100-102]. Deep TMS may be selected for patients with DOC with a damaged brain rather than the cortex.

Whether optimal rTMS parameters, such as high-frequency stimulation followed by low-frequency and bilateral stimulation, are better for DOC patient outcomes should be further explored.

### Intensity and durations

Although it is generally accepted that stimulation frequency plays a vital role in the therapeutic success of rTMS, inconsiderable attention has been paid to investigating the effects of various intensities than to the frequency-dependent effects of rTMS. The motor threshold (MT) technique is often applied for intensity selection in clinics, and the stimulation intensity is expressed as a percentage of the MT [103]. Resting motor threshold (rMT) has been classically defined as the intensity of TMS necessary to produce a motor-evoked potential (MEP). Typical intensities that vary between 80-120% of the MT for the stimulation of brain regions other than the motor cortex have been applied in clinical settings. rTM at 70-100% has been used for patients with DOC [104-107]. It remains unclear whether higher stimulation intensity protocols are better than low-intensity protocols for DOC treatment. Inadequate standardization of electric field strength may lead to variability in rTMS results and, hence, therapeutic achievement. To develop the most effective protocols, whether the impact of intensity on TMS protocols will impact therapeutic success should be further studied.

A previous study reported that increasing the duration of TMS stimulation may enhance the subsequent duration of beneficial cognitive outcomes [108]. Consistent with the CRS-R and EEG grading indices, Zhang et al. [49] found that the scores after 60 days of rTMS treatment were higher than the scores after 30 days, indicating that the difference between the two groups tended to increase over time.

### Sessions

In addition to the stimulation location, the number of sessions is another key parameter affecting the therapeutic efficacy of rTMS in patients with DOC, as they often have a long course of disease. However, little data are available on the number of sessions appropriate for patients with DOC. Generally, 1-10 sessions are used in the rTMS protocol for clinical application. For example, Naro et al. found that a single session of 10 Hz rTMS over the right DLPFC transiently improved consciousness and partially renovated the connectivity within several cortical regions in some UWS patients [46], and Piccione *et al.* reported that a single rTMS treatment improved awareness and arousal in MCS, but these effects only lasted for 6 h [1].

Interestingly, Fan et al. [31] found that 20 sessions might not be sufficient to produce full therapeutic efficacy in patients with DOC, suggesting that the number of TMS sessions is critical to the recovery of patients with DOC. Indeed, studies have shown that DOC patients receiving ≥ 10 sessions have the greatest increase in CRS-R scores, compared to improvement with ≥ 1 and < 10 sessions of TMS [54], suggesting that the number of TMS sessions received by patients has a significant impact on outcomes. Moreover, Xia et al. reported that only transient improvements in CRS-R scores were noticed in MCS patients prior to completing 10 sessions of TMS [52]. Future randomized controlled trials are needed to clarify whether 10 sessions of TMS or rTMS are the minimum number of sessions required for patients with DOC to significantly reach therapeutic efficacy.

## 6. Safety

The safety of long-term application of rTMS in patients with DOC remains unclear. TMS or rTMS for the treatment of patients with DOC is believed to be safe and has the potential to increase the CRS-R scores of some patients. Based on the systematic review and meta-analysis, serious adverse events in animals and patients have not yet been described [54], although headache, epilepsy, head tinnitus, discomfort, hearing impairment, and local skin burns have been reported in patients with other neurological disorders after rTMS application [109-111]. Accidental seizures are the most serious side effects reported with TMS to date [109, 111]; however, they have not been found among thousands of healthy and depressed subjects treated with rTMS [109], indicating that rTMS may be responsible for the later development of ‘spontaneous’ seizures in a few patients with pre-existing neuropathology. Further studies are required to examine this compilation when performing rTMS [50].

## 7. Challenges and perspectives

Although evidence has shown some clinical benefits of rTMS for VS and MCS, the study design is far from ideal, which results in inadequate data to establish recommendations regarding their use in clinical practice. First, the number of patients with DOC in most previous studies was very small. For example, Manganotti et al. [112] investigated three VS/UWS and three MCS patients and found that only one MCS patient had long-lasting behavioral and neurophysiological improvement. Naro et al. [46] treated 10 VS/UWS patients, and although significant, only 3 patients had transient, clinical improvement. Similarly, Cincotta et al. [45] reported no significant effect in 11 patients with VS/UWS after treatment. The small sample size of these studies has limitations that can compromise their drawn conclusions. Second, many studies had a small number of sessions (5-10) and a short duration of follow-up (< 3 weeks). Other parameters also affect the therapeutic efficacy of rTMS. Third, most studies used a self-controlled study before and after rTMS treatment, as it is a logistical and methodological challenge to include placebo-controlled subjects [113]. Some studies do not include control groups, and the overall quality is low, as some are case reports or prospective single-blinded studies, which limits the interpretation of the findings. Therefore, the clinical applications of rTMS for the treatment of patients with DOC remain in the exploratory stage. Further evaluation with large sample, randomized controlled trials is required to test the efficacy of rTMS in patients with DOC. In addition, a standardized rTMS protocol has not been recommended and its therapeutic effects have not been consistently recognized. The potential cause could be that different stimulation parameters may significantly impact the therapeutic efficiency. Therefore, further studies are required to develop the most effective protocols for patients with DOC by identifying optimal stimulation strategies.
